# Continuum of Care for Diabetes and Hypertension Patients During the Pandemic Era: Bridging the Gap

**DOI:** 10.21315/mjms-12-2024-989

**Published:** 2025-08-30

**Authors:** Kurnia Widyaningrum, Lilik Zuhriyah, Viera Wardhani, Achmad Rudijanto

**Affiliations:** 1 Faculty of Medicine, Universitas Brawijaya, Malang, Indonesia; 2 Department of Public Health, Faculty of Medicine, Universitas Brawijaya, Malang, Indonesia; 3 Department of Internal Medicine, Faculty of Medicine, Universitas Brawijaya, Malang, Indonesia

**Keywords:** diabetes mellitus, hypertension, continuum of care, Health Belief Model, COVID-19, continuity of care

## Abstract

**Background:**

Diabetes mellitus (DM) and hypertension (HT) are chronic diseases with an increasing prevalence in Indonesia. Continuum of care (CoC) offers a crucial management strategy, yet its implementation has faced challenges, particularly during the COVID-19 pandemic. This study examines how the Health Belief Model (HBM) construct intersects with CoC implementation in primary healthcare (PHC) settings under pandemic conditions, addressing a critical gap in understanding chronic disease management adaptations.

**Methods:**

A cross-sectional study, involving 351 DM or HT patients at nine PHC centres, was conducted between February and April 2021. Data collection included HBM-based questionnaires and structured patient surveys, analysed through descriptive statistics and path analysis.

**Results:**

Respondents were predominantly women (55.0%) aged 41–60 years old (48.1%), and with secondary or higher education (74.6%). A total of 43% reported self-medicating during the pandemic, reflecting behavioural shifts in care-seeking. Path analysis showed age consistently influenced all five HBM perceptions, while education was associated with three: perceived susceptibility, severity and benefit. Perceived benefit was the strongest positive predictor of revisit behaviour (*β* = 0.264; *P* = 0.019), whereas perceived severity had a negative effect (*β* = −0.146; *P* = 0.024). Indirectly, age and education influenced revisit behaviour through perceived benefit and susceptibility, which were the most consistent mediators.

**Conclusion:**

This study demonstrates the utility of HBM in understanding CoC adherence during health crises. The findings support the tailoring of chronic disease strategies by age and education, and enhancing CoC with digital technologies and expanded Prolanis programmes. Future research should assess the long-term impacts of HBM-based interventions on adherence and outcomes.

## Introduction

Diabetes mellitus (DM) and hypertension (HT) are chronic diseases with an increased global prevalence, specifically in low- and middle-income countries. The World Health Organization (WHO) projects an increase in DM patients in Indonesia, from 8.4 million in 2000 to 21.3 million in 2030 (1, 2). Meanwhile, the prevalence of HT in the population aged > 18 years old has also increased by 34.1% since 2013 (3). The increase is not only a burden on the health system but also has a serious impact on the quality of life of individuals and increases the risk of cardiovascular complications. This challenge has led to the development of the continuum of care (CoC) method as a key strategy for managing chronic diseases. The CoC is a structured and sustainable health method that includes cross-disciplinary coordination, from prevention to long-term disease management (4). In the context of DM and HT, CoC enables integrated access to medical care, ongoing education and routine monitoring, potentially improving disease management, preventing complications and enhancing the life quality of patients (5, 6).

The COVID-19 pandemic created new challenges in implementing the CoC. Patients with DM and HT face higher risks of complications and mortality related to COVID-19 (7, 8), and mobility restrictions and fear of virus transmission hinder access to routine health services. This situation is compounded by stigma and social isolation, which negatively impact health management and the mental well-being of patients (9, 10). The Health Belief Model (HBM) offers an important theoretical framework suitable for understanding and overcoming these barriers. HBM analyses the effect of patients’ perceptions of disease risk, benefits of care and barriers (11). This understanding is important for designing effective interventions and improving patient adherence to the CoC programme. Therefore, this study aimed to fill the gap by analysing the relationship among social factors, demographic factors, and perceptions of primary healthcare (PHC) revisit behaviour in DM and HT patients in Malang Raya, Indonesia.

Although the COVID-19 pandemic has subsided, the importance of CoC in managing DM and HT remains relevant. Continuity of coordinated care allows for regular monitoring, management of complication risks and appropriate early intervention (12, 13). This study also focused on primary care, which is the frontline of chronic disease management. Therefore, a unique perspective on the challenges and opportunities in maintaining continuity of care at the community level was provided.

These results are expected to contribute to the development of more targeted strategies for improving continuity of care and achieving optimal therapeutic targets for DM and HT patients. The results will also help policymakers and healthcare providers design interventions that are more effective and responsive to the needs of patients with chronic diseases.

## Methods

### Research Design

A cross-sectional design was used to analyse factors that influence care sustainability for DM and HT patients in Malang Raya. The research was conducted over three months, from February to April 2021.

### Population and Sample

The study population comprised individuals diagnosed with DM and/or HT who visited nine selected PHC centres in Malang Raya during the data collection period. A consecutive sampling method was used, in which all eligible patients who met the inclusion criteria and agreed to participate were recruited sequentially as they presented at the PHCs. This approach was chosen for its practicality in real-world settings and its capacity to ensure representative inclusion within the designated timeframe.

The minimum sample size required for the study was determined using Cochran’s formula, which is commonly used in cross-sectional studies involving large populations. Based on the standard assumptions for proportion estimates, the calculated sample size was approximately 385 respondents. Initially, 500 patients were approached. After applying the inclusion and exclusion criteria and removing incomplete or invalid responses, 351 respondents were retained for the final analysis. Although this number is slightly below the estimated minimum, it remains within an acceptable range and is considered sufficient for analysis.

### Research Instrument

To ensure the quality and rigour of the instrument, the questionnaire underwent a series of validation and reliability assessments. The initial questionnaire comprised 31 items developed based on six HBM constructs and one behavioural outcome. Construct validity was tested using exploratory factor analysis (EFA). Based on the factor loading threshold of ≥ 0.60, three items were excluded due to low loading values. The final measurement model retained 28 valid indicators, all demonstrating statistically significant loadings (*P* < 0.001), thus confirming convergent validity.

Reliability was evaluated using Cronbach’s alpha for each construct. All constructs showed strong internal consistency, with alpha values ranging from 0.870 (health motivation) to 0.976 (perceived benefit), exceeding the minimum threshold of 0.70. These results confirm that the measurement instrument was both valid and reliable for further analysis.

The final questionnaire consisted of 28 items designed to capture a range of variables relevant to the study. These included: i) demographic factors, such as age, gender and educational attainment; ii) social factors, such as employment type and income level; iii) patient perceptions, including perceived susceptibility, severity, benefits, barriers and health motivation; and iv) health behaviours, such as dietary adherence, physical activity and participation in chronic disease management programmes (Prolanis). Clinical indicators, such as fasting blood sugar and blood pressure results, were also recorded.

All items measuring perception and motivation were assessed using a 5-point Likert scale, ranging from 1 (strongly disagree) to 5 (strongly agree). Higher scores indicated stronger agreement with statements related to health beliefs, enabling quantification of patients’ perceptions and behavioural tendencies in accordance with the HBM framework.

### Data Collection Procedure

Two data collection strategies were implemented based on the respondents’ literacy levels. Patients who were literate completed the questionnaire independently, while those who faced difficulties in reading or writing or who were of advanced age were assisted through structured interviews conducted by surveyors. At each PHC site, two trained surveyors were assigned to administer the questionnaires. All surveyors received comprehensive training prior to data collection to ensure consistency, ethical compliance and adherence to standardised procedures.

### Data Analysis

Data analysis was conducted in three stages. First, descriptive statistics were used to summarise respondent characteristics and behavioural patterns. Second, the measurement model was assessed through EFA and Cronbach’s alpha, as previously described. Third, path analysis was performed using AMOS software to examine the direct and indirect effects of demographic variables and HBM constructs on revisit behaviour, in alignment with the study framework.

Prior to path analysis, the model fit was evaluated to ensure structural validity. The model demonstrated good fit across multiple indices: chi-square/degrees of freedom (CMIN/DF) = 1.685; root mean square error of approximation (RMSEA) = 0.044; goodness of fit index (GFI) = 0.991; adjusted goodness of fit index (AGFI) = 0.939; Tucker-Lewis index (TLI) = 0.969; and comparative fit index (CFI) = 0.994. The chi-square test yielded a non-significant result (*P* = 0.063), confirming that the hypothesised model adequately represented the observed data.

## Results

The demographic characteristics of the chronic disease patients showed significant differences between the routine control and self-medicating groups (Table 1). The routine control group was dominated by women (61.00%) and patients aged 41–80 years old (93.50%), while the self-medicating group had a higher proportion of men (52.98%) and patients under 40 years old (29.14%). In terms of socioeconomic status, patients with low income (< IDR1,000,000) were more likely to self-medicate (40.4%) than the routine control group (30.0%), indicating that economic factors play a role in healthcare decision-making during the COVID-19 pandemic. Table 2 reveals significant differences in health perceptions between the two groups. Patients who underwent routine check-ups had higher perceived susceptibility (3.90) and health motivation (3.98) scores than the self-medicating group (3.42 and 3.45, respectively), both in the high and moderate categories. Both groups had high perceived severity and perceived benefits, but with higher scores in the routine control group (3.84 and 4.14) compared to self-medicating (3.62 and 3.61). Perceived barriers were low in both groups, with slightly lower scores in the routine control group (2.26 vs 2.43), indicating that routine control patients had higher awareness of disease susceptibility and stronger health motivation.

A comparison of the clinical and behavioural characteristics of patients was made; there was a striking difference in participation in the Prolanis programme, where 72.50% of routine control patients were involved in this programme compared to only 28.48% of the self-medicating group (Table 3). Routine control patients also showed better health behaviours, such as diabetes diet compliance (48.50% vs 39.07%) and higher exercise frequency (33.50% exercised ≥ 3 times/week vs 19.87%). The distribution of disease duration showed that patients with long-term HT (> 5 years) were more likely to have regular check-ups (30.00% vs 15.89%), indicating that long-term experience with chronic conditions may increase awareness of the importance of regular medical supervision.

Tables 4 and 5 present the detailed path analysis results highlighting patients’ health perceptions and treatment-seeking behaviours, with an emphasis on the mediating roles of HBM constructs. The path analysis revealed that age was the only demographic variable significantly associated with all five HBM perceptions: perceived susceptibility, severity, benefit, barrier and health motivation. All associations were positive except for perceived barriers, which showed a negative relationship. Education influenced three perceptions, namely, susceptibility, severity, and benefit, while gender and income each showed significance for only one construct, namely perceived benefit and perceived susceptibility, respectively. Occupation did not significantly influence any perception variable. These findings are clearly demonstrated in Table 4, which presents the direct effect coefficients from the path analysis, showing the magnitude and statistical significance of each relationship between demographic variables and health belief components.

Table 5 presents the indirect effects of sociodemographic factors on revisit behaviour through various components of the HBM, looking at the mediating pathways from patient characteristics to ultimately influencing healthcare decisions. Among the HBM predictors of revisit behaviour, perceived benefit showed the strongest positive effect (*β* = 0.264; *P* = 0.019), followed by perceived susceptibility (*β* = 0.154; *P* = 0.079, marginally significant). In contrast, perceived severity showed a significant negative association (*β* = −0.146; *P* = 0.024), suggesting that severity alone does not necessarily motivate patients to return for care. Health motivation, perceived barriers and cues to action did not show significant direct effects.

## Discussion

This study provides important information on factors contributing to the sustainability of care for DM and HT patients in Malang Raya. The results showed the complexity of the challenges faced in the management of these chronic diseases. These results are relevant for the pandemic period and offer valuable lessons for improving chronic disease management in the post-pandemic era.

### Demographic Characteristics and Treatment Behaviour of Chronic Disease Patients During the COVID-19 Pandemic

This study reveals a distinctive pattern in the treatment behaviour of chronic disease patients in Indonesia during the COVID-19 pandemic. The data showed that the majority of respondents (56.7%) chose to self-medicate, while 43.3% continued to have routine check-ups at health facilities. This reflects a significant shift in health behaviour during the global health crisis, where limited access to and fear of exposure to the virus are major considerations for chronic patients (14, 15).

An interesting finding is seen in the gender distribution, where female patients are more likely to have routine check-ups (61%) than male patients (39%). In contrast, the self-medicating group is dominated by men (52.98%). This indicates that women in Indonesia have a higher tendency to comply with prescribed treatment regimens, which is in line with previous studies showing gender differences in health-seeking behaviour. The Indonesian cultural context, which positions women as guardians of family health, may contribute to this tendency (16, 17).

In terms of age, there was a striking difference where the routine control group was dominated by patients aged 41–80 years old (93.50%), while the self-medicating group had a higher proportion of patients under 40 years old (29.14% vs 5.50%). This phenomenon can be explained by different risk perceptions between age groups, where younger patients may feel that they have a lower risk of chronic disease complications or are more confident in managing their own treatment. On the other hand, older patients may be more aware of the risk of complications and the importance of professional medical supervision (18).

These results showed the importance of a gender and age-sensitive method in designing chronic disease management programmes. Interventions targeted at this demographic group may be more effective in improving long-term health outcomes.

### Influence of Socioeconomic Factors on Health Behaviour During the Pandemic

Analysis of socioeconomic status revealed an interesting pattern, where patients with lower incomes (< IDR1,000,000) were more likely to self-medicate (40.4% vs 30.0%). This indicates that economic constraints are an important factor influencing treatment decisions, especially in the context of a health crisis. Transportation costs, health care costs, and potential loss of income due to time spent on medical visits may be significant considerations for low-income groups (19).

The path analysis results support this finding by showing that income has a significant negative effect on perceived susceptibility (*β* = −0.147, *P* = 0.041). This means that the lower a person’s income, the lower their perceived susceptibility to disease, which in turn affects the decision to self-medicate. This finding reflects the gap in health literacy and access to information, which is still a challenge in developing countries, such as Indonesia. Education level also plays an important role, with the path analysis results showing a significant positive effect on perceived benefit (*β* = 0.199, *P* = 0.008), perceived severity (*β* = 0.183, *P* = 0.010) and perceived susceptibility (*β* = 0.157, *P* = 0.007). This suggests that higher education is correlated with a better understanding of the benefits of treatment and the severity of the disease. This phenomenon has important implications for health education strategies in Indonesia and other Southeast Asian countries with similar demographic characteristics (20, 21).

### The Role of Health Perceptions and Motivation in Treatment Adherence

Differences in health perceptions between the two groups were substantial, with routine check-up patients scoring higher on perceived susceptibility (3.90 vs 3.42) and health motivation (3.98 vs 3.45). Path analysis confirmed that perceived benefit had a significant positive influence on adherence (*β* = 0.264, *P* = 0.019), while perceived severity had a paradoxical negative effect (*β* = −0.146, *P* = 0.024). This suggests that while disease severity awareness is important, it does not necessarily translate into adherence without an understanding of treatment benefits. In Indonesia, where the collectivist culture emphasises community norms and shared health experiences, these perceptions can be shaped by familial and social influences. Consequently, individuals who are embedded in health-aware communities may demonstrate greater motivation for regular health check-ups and adherence to treatment (14).

These findings highlight the need for intervention strategies that reinforce the advantages of regular medical care rather than focusing solely on disease severity. In Indonesia’s collectivist society, health decisions are often influenced by social norms and shared experiences. This finding aligns with previous literature, which suggests that health motivations are often fuelled by positive reinforcement of the outcomes of adherence rather than by fear of disease complications (15). The COVID-19 pandemic further complicated this dynamic, as patients weighed the risk of virus exposure against the need for chronic disease management, necessitating adaptable healthcare communication approaches.

### The Role of Prolanis in Chronic Disease Management During the Pandemic

This study highlights the effectiveness of Prolanis in ensuring continuity of care, even during crises. Patients enrolled in Prolanis were significantly more likely to adhere to scheduled check-ups (72.50%) compared to those who self-medicated (28.48%). The structured nature of Prolanis, which integrates education, routine monitoring and community support, likely played a key role in maintaining patient motivation and adherence to medical care. The strong association between Prolanis participation and adherence to healthier behaviours, such as dietary compliance (48.50% vs 39.07%) and exercise (33.50% vs 19.87%), underscores its potential as a scalable model for chronic disease management. This result is consistent with the report of Khoe et al. (22) and Anwar et al. (23) that community programmes are important in the management of chronic disease.

As a unique initiative within Indonesia’s National Health Insurance, Prolanis offers a comprehensive approach to chronic disease care through a combination of patient education, routine health monitoring, and peer support. During the pandemic, the community-driven support system may have been instrumental in sustaining patient engagement, particularly in overcoming psychological and logistical barriers to care (24). These findings suggest that integrated care models, such as Prolanis, could serve as effective interventions for chronic disease management, not only in Indonesia but also in other Southeast Asian countries facing similar healthcare challenges. Expanding such programmes may enhance healthcare resilience during future public health emergencies.

### Adapting the HBM in the Indonesian Context

This study provides critical insights into the adaptation of HBM within the Indonesian cultural context. Unlike in Western populations, where perceived susceptibility is a key driver of healthcare behaviour, Indonesian patients exhibited a stronger reliance on perceived benefit as the primary determinant of adherence (*β* = 0.264, *P* = 0.019). This suggests that tangible treatment outcomes, rather than risk awareness, play a more dominant role in motivating patient decisions (25, 26). These findings highlight the need for restructuring health messaging strategies, shifting from risk-focused approaches to those that emphasise the direct advantages of treatment adherence in improving long-term health outcomes.

The inverse relationship between perceived severity and healthcare adherence (*β* = −0.146, *P* = 0.024) presents an intriguing paradox. This may reflect a fatalistic attitude, in which heightened awareness of disease severity, without a corresponding understanding of treatment efficacy, leads to inaction or a preference for alternative medicine. Cultural context plays a crucial role in shaping these perceptions. The complexity of Indonesian society, with its diverse cultural beliefs and practices, influences how health information is received and processed. For example, cultural beliefs are embedded in daily life and can either support or hinder access to healthcare, thereby affecting health behaviours, such as medication adherence (27, 28). Additionally, age played a crucial role, with older patients demonstrating a more accurate understanding of chronic disease risks and benefits, reinforcing the need for age-specific interventions in Indonesia’s healthcare strategy. These findings underscore the importance of tailoring chronic disease management programmes to better align with patient perceptions and cultural dynamics to enhance long-term adherence.

Based on these results, several practical implications were proposed. First, patient programmes need to be adjusted to age and education levels to increase effectiveness. Second, strengthening Prolanis with telemedicine integration can overcome the access barriers caused by the pandemic. Third, strategies to reduce barriers to routine visits, including overcoming fears of disease transmission in health facilities, need to be developed. Fourth, health campaigns that focus on the importance of effective self-management should be enhanced. Finally, leveraging digital technology for remote monitoring and ongoing support can be an innovative solution in DM and HT management.

### Limitations and Future Directions

While this study provides valuable insights, several limitations must be acknowledged. The cross-sectional design limits causal interpretations of the relationships observed between variables. The temporal nature of the associations cannot be definitively established, and future longitudinal studies would provide stronger evidence for the causal pathways proposed in this research.

The geographical focus on Malang Raya may restrict the generalisability of findings to other regions in Indonesia or other countries with different healthcare systems and cultural contexts. The unique characteristics of this region, including its healthcare infrastructure and population demographics, may not be representative of other settings.

Future research should prioritise longitudinal studies to evaluate the sustainability and long-term impact of HBM-based interventions on chronic disease management outcomes. Key areas for investigation include assessing the effectiveness of digital health innovations such as telemedicine and mobile health applications, adapting chronic disease management programmes across different cultural contexts in Southeast Asia, developing culturally tailored health messaging strategies that emphasise treatment benefits, and examining the cost-effectiveness of community-based programmes like Prolanis compared to traditional individual-based care models to inform healthcare policy decisions and resource allocation strategies.

## Conclusion

This study highlights the critical role of patient perceptions and socioeconomic factors in shaping healthcare-seeking behaviour among individuals with chronic diseases during the COVID-19 pandemic. The findings demonstrate that perceived benefits, rather than disease severity, are the strongest predictors of adherence to routine healthcare visits. Additionally, demographic characteristics, particularly age and education level, significantly influence these perceptions, underlining the need for targeted intervention strategies.

The effectiveness of community-based programmes such as Prolanis in maintaining continuity of care was evident, with higher participation rates associated with improved adherence to medical check-ups and healthier lifestyle behaviours. These findings suggest that integrating structured disease management programmes and leveraging digital health solutions, such as telemedicine, can enhance patient engagement and reduce healthcare disparities.

To optimise chronic disease management in post-pandemic settings, policymakers and healthcare providers should focus on improving health literacy, reducing perceived barriers to care and emphasising the tangible benefits of treatment adherence. Future research should explore the long-term impact of HBM-based interventions and assess the effectiveness of digital innovations in ensuring sustained healthcare access for chronic disease patients. By addressing these critical factors, healthcare systems can develop more effective patient-centred strategies to improve chronic disease management, ultimately leading to better health outcomes and a more resilient healthcare infrastructure.

## Figures and Tables

**Table 1:** Patient characteristics: comparison between regular medical control and self-medicating groups

Characteristic	Category	Total		Regular medical control		Self-medicating	

		*n*	%	*n*	%	*n*	%
Gender	Male	158	45.0	78	39.0	80	53.0
	Female	193	55.0	122	61.0	71	47.0

Age (years)	≤ 20	7	2.0	1	0.5	6	4.0
	21–40	48	13.7	10	5.0	38	25.2
	41–60	169	48.1	98	49.0	71	47.0
	61–80	125	35.6	89	44.5	36	23.8
	> 80	2	0.6	2	1.0	0	0

Education	Elementary school	50	14.2	30	15.0	20	13.2
	Junior high school	36	10.2	19	9.5	17	11.3
	Senior high school	102	29.1	54	27.0	48	31.8
	Undergraduate	129	36.8	74	37.0	55	36.4
	Post graduate	31	8.8	21	10.5	10	6.6
	Others	3	0.9	2	1.0	1	0.7

Income (IDR)	< 1,000,000	121	34.5	60	30.0	61	40.4
	1,000,000–3,000,000	84	23.9	57	28.5	27	17.9
	3,000,000–5,000,000	101	28.8	65	32.5	36	23.8
	> 5,000,000	45	12.8	18	9.0	27	17.9

Occupation	Health workers	29	8.3	16	8.0	13	8.6
	Non-health workers	257	73.2	142	71.0	115	76.2
	Not working	65	18.5	42	21.0	23	15.2

**Table 2:** Comparison of perceptions between regular medical control and self-medicating patients

Perception	Regular medical control		Self-medicating	

	Average	Category	Average	Category
Perceived susceptibility	3.90	High	3.42	Moderate
Perceived severity	3.84	High	3.62	High
Perceived benefit	4.14	High	3.61	High
Perceived barriers	2.26	Low	2.43	Low
Health motivation	3.98	High	3.45	Moderate

**Table 3:** Comparison of clinical characteristics and behaviours between patient groups

Clinical characteristics and behaviours	Regular medical control		Self-medicating	

	*n*	%	*n*	%
**Duration of DM diagnosis**			F	P
Non-diabetic	82	41.4	59	39.3
< 1 year	30	15.2	35	23.3
1–5 years	39	19.7	31	20.7
> 5 years	47	23.7	25	16.7
**Duration of HT diagnosis**				
Non-hypertensive	45	22.5	60	39.7
< 1 year	44	22.0	37	24.5
1–5 years	51	25.5	30	19.9
> 5 years	60	30.0	24	15.9
**Diet compliance**				
Yes	97	49.0	59	39.3
No	101	51.0	91	60.7
**Join the Prolanis group**				
Yes	145	72.5	43	28.5
No	55	27.5	108	71.5
**Exercise frequency**				
< 3 times/week	131	66.1	118	79.7
3–5 times/week	52	26.3	23	15.6
> 5 times/week	15	7.6	7	4.7

**Table 4:** Path analysis direct effect

Independent variable	Dependent variable	Direct effect coefficient	Standardised direct effect coefficient	*P*-value
**Gender**				
Gender	Health motivation	1.140	0.076	0.124
Gender	Perceived barrier	−0.835	−0.076	0.245
Gender	Perceived benefit	1.241	0.149	0.015*
Gender	Perceived severity	0.597	0.104	0.066
Gender	Perceived susceptibility	0.381	0.086	0.104
**Age**				
Age	Health motivation	0.079	0.151	0.009*
Age	Perceived barrier	−0.077	−0.199	0.007*
Age	Perceived benefit	0.059	0.200	0.006*
Age	Perceived severity	0.037	0.181	0.003*
Age	Perceived susceptibility	0.030	0.195	0.008*
**Education**				
Education	Health motivation	0.666	0.112	0.082
Education	Perceived barrier	0.061	0.014	0.831
Education	Perceived benefit	0.660	0.199	0.008*
Education	Perceived severity	0.419	0.183	0.010*
Education	Perceived susceptibility	0.278	0.157	0.007*
**Income**				
Income	Health motivation	−0.662	−0.094	0.280
Income	Perceived barrier	−0.529	−0.102	0.120
Income	Perceived benefit	−0.353	−0.090	0.173
Income	Perceived severity	−0.263	−0.097	0.270
Income	Perceived susceptibility	−0.308	−0.147	0.041*
**Occupation**				
Occupation	Health motivation	2.212	0.116	0.071
Occupation	Perceived barrier	−0.061	−0.004	0.998
Occupation	Perceived benefit	0.448	0.042	0.427
Occupation	Perceived severity	0.211	0.029	0.682
Occupation	Perceived susceptibility	0.620	0.110	0.149
**Other variables**				
Cues to action	Action	0.037	0.135	0.090
Health motivation	Action	−0.020	−0.098	0.297
Perceived barrier	Action	−0.006	−0.020	0.751
Perceived benefit	Action	0.097	0.264	0.019*
Perceived severity	Action	−0.077	−0.146	0.024*
Perceived susceptibility	Action	0.106	0.154	0.079

* significant at *P* < 0.05

**Table 5:** Path analysis indirect effects

Independent variable	Dependent variable	Mediation path	Standardised indirect effect	*P*-value	Effect direction
**Gender**					
Gender	Action	Health motivation → Action	−0.007	0.124	Negative
Gender	Action	Perceived barrier → Action	0.002	0.245	Positive
Gender	Action	Perceived benefit → Action	0.039	0.015*	Positive
Gender	Action	Perceived severity → Action	−0.015	0.066	Negative
Gender	Action	Perceived susceptibility → Action	0.013	0.104	Positive
**Age**					
Age	Action	Health motivation → Action	−0.015	0.009*	Negative
Age	Action	Perceived barrier → Action	0.004	0.007*	Positive
Age	Action	Perceived benefit → Action	0.053	0.006*	Positive
Age	Action	Perceived severity → Action	−0.026	0.003*	Negative
Age	Action	Perceived susceptibility → Action	0.030	0.008*	Positive
**Education**					
Education	Action	Health motivation → Action	−0.011	0.082	Negative
Education	Action	Perceived barrier → Action	−0.0003	0.831	Negative
Education	Action	Perceived benefit → Action	0.053	0.008*	Positive
Education	Action	Perceived severity → Action	−0.027	0.010*	Negative
Education	Action	Perceived susceptibility → Action	0.024	0.007*	Positive
**Income**					
Income	Action	Health motivation → Action	0.009	0.280	Positive
Income	Action	Perceived barrier → Action	0.002	0.120	Positive
Income	Action	Perceived benefit → Action	−0.024	0.173	Negative
Income	Action	Perceived severity → Action	0.014	0.270	Positive
Income	Action	Perceived susceptibility → Action	−0.023	0.041*	Negative
**Occupation**					
Occupation	Action	Health motivation → Action	−0.011	0.071	Negative
Occupation	Action	Perceived barrier → Action	0.0001	0.998	Positive
Occupation	Action	Perceived benefit → Action	0.011	0.427	Positive
Occupation	Action	Perceived severity → Action	−0.004	0.682	Negative
Occupation	Action	Perceived susceptibility → Action	0.017	0.149	Positive

* significant at *P* < 0.05;

The standardised indirect effect (*β*) values for specific mediation paths were calculated based on the direct path coefficients from independent variables to mediators and from mediators to dependent variables

## References

[1] Ogurtsova K, da Rocha Fernandes JD, Huang Y, Linnenkamp U, Guariguata L, Cho NH (2017). IDF Diabetes Atlas: global estimates for the prevalence of diabetes for 2015 and 2040. Diabetes Res Clin Pract.

[2] Soelistijo SA, Lindarto D, Decroli E, Permana H, Sucipto KW, Kusnadi Y (2019). Pedoman pengelolaan dan pencegahan diabetes melitus tipe 2 dewasa di Indonesia 2019. [Internet].

[3] Debora C, Tolimba C, Palunggi S, Siregar D, Harefa L (2023). Risk Factors for hypertension among adults living in a rural area, Minahasa. J Keperawatan Indones.

[4] Liao K, Lin K-C, Chiou S-J (2021). Self-efficacy remains a vital factor in reducing the risk of dialysis in type 2 diabetes care. Medicine.

[5] Sumner J, Bundele A, Chong LS, Teng GG, Kowitlawakul Y, Mukhopadhyay A (2022). Continuing chronic care services during a pandemic: results of a mixed-method study. BMC Health Serv Res.

[6] Chan K-S, Wan EY-F, Chin W-Y, Cheng WH-G, Ho MK, Yu EY-T (2021). Effects of continuity of care on health outcomes among patients with diabetes mellitus and/or hypertension: a systematic review. BMC Fam Pract.

[7] Wu ZH, Tang Y, Cheng Q (2021). Diabetes increases the mortality of patients with COVID-19: a meta-analysis. Acta Diabetol.

[8] Ghilari YED, Iskandar A, Wiratama BS, Hartopo AB (2022). Joint effect of diabetes mellitus and hypertension on COVID-19 in-hospital mortality stratified by age group and other comorbidities: a cohort retrospective study using hospital-based data in Sleman, Yogyakarta. Healthcare.

[9] Ramaci T, Barattucci M, Ledda C, Rapisarda V (2020). Social stigma during COVID-19 and its impact on HCWs outcomes. Sustainability.

[10] Bhanot D, Singh T, Verma SK, Sharad S (2021). Stigma and discrimination during COVID-19 pandemic. Front Public Heal.

[11] Mohamed RA, Mohammed Abd-Elghany S, Mohammad Abd Elrhman SH, Taref NN (2022). Effect of applying health belief model on type 2 diabetic patients’ knowledge, self-care practices and health beliefs. Egypt J Health Care.

[12] Gabert R, Ng M, Sogarwal R, Bryant M, Deepu RV, McNellan CR (2017). Identifying gaps in the continuum of care for hypertension and diabetes in two Indian communities. BMC Health Serv Res.

[13] Carman KL, Workman TA (2017). Engaging patients and consumers in research evidence: applying the conceptual model of patient and family engagement. Patient Educ Couns.

[14] Stickley A, Matsubayashi T, Sueki H, Ueda M (2020). COVID-19 preventive behaviors among people with anxiety and depression: findings from Japan. Public Health.

[15] Karyono DR, Wicaksana AL (2020). Current prevalence, characteristics, and comorbidities of patients with COVID-19 in Indonesia. J Community Empower Health.

[16] Gonzalez TN, Steffens T, Perim LF, Ritta M, Silva DC, Machado KP (2023). Symptom patterns of long COVID and chronic illness: a cross-sectional analysis of the SulCovid-19 study. [Preprint].

[17] Rachmawati E, Listiowati E, Kurniawan DW, Suraya I, Ahsan A, Nurmansyah MI (2021). Significance of chronic diseases and smoking behavior in the development of acute respiratory distress syndrome among hospitalized COVID-19 patients in Indonesia. Asia Pac J Public Health.

[18] Abraham SA, Agyare DF, Yeboa NK, Owusu-Sarpong AA, Banulanzeki ES, Doku DT (2023). The influence of COVID-19 pandemic on the health seeking behaviors of adults living with chronic conditions: a view through the health belief model. J Prim Care Community Health.

[19] Meisters R, Putrik P, Westra D, Bosma H, Ruwaard D, Jansen M (2023). Two sides of the same coin? Absolute income and perceived income inadequacy as social determinants of health. Int J Equity Health.

[20] Guo A, Jin H, Mao J, Zhu W, Zhou Y, Ge X (2023). Impact of health literacy and social support on medication adherence in patients with hypertension: a cross-sectional community-based study. BMC Cardiovasc Disord.

[21] Liu YB, Hou P, Xue HP, Mao XE, Li YN (2019). Social support, health literacy, and health care utilization among older adults. Asia Pac J Public Health.

[22] Khoe LC, Wangge G, Soewondo P, Tahapary DL, Widyahening IS (2020). The implementation of community-based diabetes and hypertension management care program in Indonesia. PLoS ONE.

[23] Anwar SA, Arsyad DS, Dwinata I, Ansar J, Rachmat M (2020). Quality life of PROLANIS participants using WHOQOL BREF Indonesian version: a community in primary health care. Enferm Clin.

[24] Stevenson C, Wakefield JRH, Felsner I, Drury J, Costa S (2021). Collectively coping with coronavirus: local community identification predicts giving support and lockdown adherence during the COVID-19 pandemic. Br J Soc Psychol.

[25] Bakhshi S, Heidari S, Zanjirani S, Zakeri MA (2023). The effect of health belief model-based education on empowering cardiovascular patients for medication adherence. J Nurs Midwifery Sci.

[26] Suryatinah Y, Athiyah U, Mohd Ali AB, Zairina E (2023). The development and validation of the Indonesian Insulin Adherence Influence Factor Questionnaire (IIAIFQ). J Farm Ilmu Kefarmasian Indones.

[27] Vionalita G, Kusumaningtiar DA, Angkasa D (2023). Relationship between health belief model constructs and smoking behavior among school-age adolescents in Indonesia: a cross-sectional study. Public Health Indones.

[28] Shahabi N, Hosseini Z, Ghanbarnejad A, Aghamolaei T (2024). Predictors of treatment adherence in patients with type 2 diabetes: a cross-sectional study in Southern Iran based on Pender’s Health Promotion Model using structural equation modelling. BMJ Open.

